# Nanocomposites for Food Packaging Applications: An Overview

**DOI:** 10.3390/nano11010010

**Published:** 2020-12-23

**Authors:** Jawad Sarfraz, Tina Gulin-Sarfraz, Julie Nilsen-Nygaard, Marit Kvalvåg Pettersen

**Affiliations:** Nofima-Norwegian Institute of Food, Fisheries and Aquaculture Research, P.O. Box 210, NO-1431 Ås, Norway; tina.gulin-sarfraz@nofima.no (T.G.-S.); julie.nilsen-nygaard@nofima.no (J.N.-N.); marit.kvalvag.pettersen@nofima.no (M.K.P.)

**Keywords:** nanocomposites, food packaging, barrier, antimicrobial, active, migration, toxicity, consumers, recyclability

## Abstract

There is a strong drive in industry for packaging solutions that contribute to sustainable development by targeting a circular economy, which pivots around the recyclability of the packaging materials. The aim is to reduce traditional plastic consumption and achieve high recycling efficiency while maintaining the desired barrier and mechanical properties. In this domain, packaging materials in the form of polymer nanocomposites (PNCs) can offer the desired functionalities and can be a potential replacement for complex multilayered polymer structures. There has been an increasing interest in nanocomposites for food packaging applications, with a five-fold rise in the number of published articles during the period 2010–2019. The barrier, mechanical, and thermal properties of the polymers can be significantly improved by incorporating low concentrations of nanofillers. Furthermore, antimicrobial and antioxidant properties can be introduced, which are very relevant for food packaging applications. In this review, we will present an overview of the nanocomposite materials for food packaging applications. We will briefly discuss different nanofillers, methods to incorporate them in the polymer matrix, and surface treatments, with a special focus on the barrier, antimicrobial, and antioxidant properties. On the practical side migration issues, consumer acceptability, recyclability, and toxicity aspects will also be discussed.

## 1. Introduction

**Figure 1:**
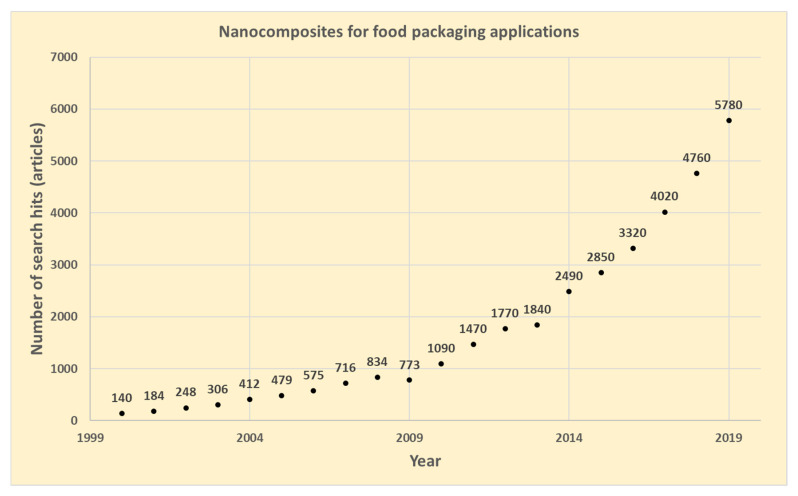
Number of annual publications related to nanocomposites for food packaging applications (searching with Google Scholar on 18 June 2020 with the following keywords: nanocomposites, food packaging).

Nanotechnology involves the characterization, fabrication, and/or manipulation of structures, devices, or materials that have at least one dimension (or contain components with at least one dimension) that is approximately 1–100 nm in length [1]. Public opinion varies about the use of nanotechnology in the food sector. While the public is predominantly against the direct use of nanomaterials in food, the use of nanotechnology in “out-of-food” applications is mostly acceptable [2,3,4]. There has been an increasing interest in nanocomposites for food packaging applications during the last two decades. From 2010, a strong increase has been observed in the number of publications with the number per year increasing more than five-fold during the period 2010–2019, as shown in Figure 1 (source: Google Scholar, keywords: nanocomposites, food packaging). The research area, however, is quite broad, where reports related to the introduction of different functionalities and improved properties of packaging materials by utilizing nanotechnology have been published. Since the barrier, mechanical, and thermal properties are the most important for food packaging applications, a lot of research effort has been done within this area [5,6,7,8,9,10,11]. Similarly, among others, active (antimicrobial/antioxidant) [12,13,14], antifouling [15,16], encapsulation [17], and sensing [18,19,20] functionalities via applying nanotechnology have also been reported in the literature. 

**Figure 2:**
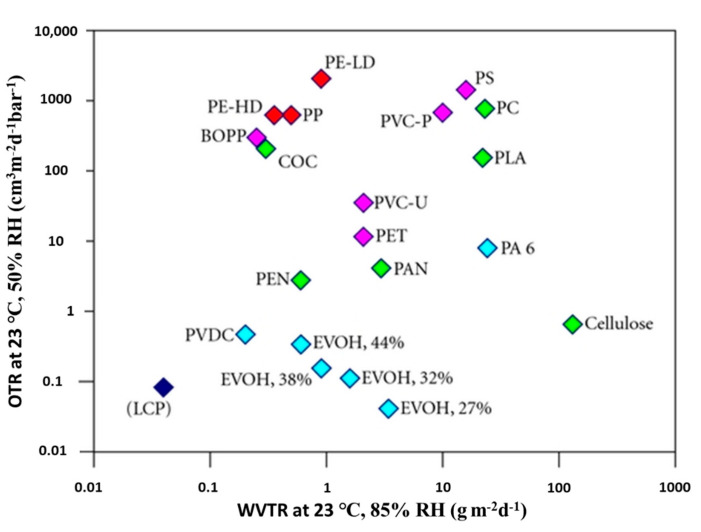
Oxygen permeability (OP) and water vapor transmission rate (WVTR) normalized to a thickness of 100 μm for different polymers. Source: Fraunhofer IVV [7,23].

Different polymers exhibit different barrier properties, for example, polyethylene terephthalate (PET) offers a good barrier to oxygen compared to high-density polyethylene (HDPE). On the other hand, HDPE provides a better barrier against water vapor compared to PET, as shown in Figure 2 [21]. Generally, the barrier properties of the polymer are affected by many factors, including the degree of branching, hydrogen bonding, polarity, cross-linking, and degree of crystallinity [1]. Furthermore, the permeability to one migrant can be affected in the presence of another, for example, the oxygen barrier properties of ethylene vinyl alcohol (EVOH) are significantly reduced in high humidity conditions due to polymer swelling and plasticization [22]. 

**Figure 3:**
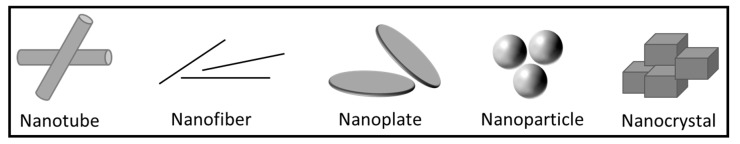
Examples of various nanoscale filler morphologies used in polymer nanocomposites. Adapted from [37].

Multi-layered structures comprising various polymers with distinct properties are used in food packaging applications to obtain appropriate mechanical, barrier, and sealing properties. There is added cost associated with these complex structures due to the use of supplementary additives and adhesives (in the case of laminates). Furthermore, these multi-layered structures are generally not recyclable. Consequently, there is a strong drive in the industry to develop packaging solutions that target sustainable development, green consumerism, and the circular economy, which pivots around the recyclability of the packaging materials. The aim is to achieve high recycling rates while maintaining the desired barrier and mechanical properties, in addition to safety aspects, such as migration requirements. In this domain, packaging materials in the form of polymer nanocomposites (PNCs) can offer the desired functionalities while maintaining their recyclability. PNCs are formed by dispersing an inert, nanoscale filler throughout a polymeric matrix [1]. The most common filler materials include clay and silicate nanoplatelets, silica nanoparticles [24,25], carbon nanotubes [26,27], graphene [28], starch nanocrystals [29], cellulose-based nanofibers or nanowhiskers [30,31], chitin or chitosan nanoparticles [32], and other inorganics [33]. In addition, it has been reported in the literature that PNC show improved strength [34], flame resistance [35], and thermal properties [36]. Typical morphologies of nanofillers are shown in Figure 3. 

In accordance with the green shift, industry will gradually move toward producing packaging materials from bio-based resources, thus limiting the production and consumption of fossil-based plastics. With the increased focus on sustainability, food packaging materials from renewable resources are increasingly demanded from consumers, food producers, and retailers, thereby gaining market shares from conventional plastic packaging materials. However, biobased and biodegradable materials from either synthetic biopolymers, such as polylactic acids (PLA), polyhydroxyalkanoates (PHA), and poly-(butylene succinate) (PBS), or natural biopolymers, such as starch, chitosan, alginate, or gelatin, generally have inferior barrier and mechanical properties compared to conventional fossil-based plastics [38]. Furthermore, their brittleness, low heat distortion temperature, and poor resistance to deformation during processing have put restraints on their possible applications [39]. On this basis, great research effort has been put into the development of biomaterials with desired properties to meet the demands of different food products with regard to e.g., the gas barrier (O_2_, CO_2_, H_2_O), water and fat resistance, and mechanical properties, as well as to ensure efficiency in industrial processing and runability on packaging machinery. The use of nanotechnology shows promising results for obtaining biomaterials that fulfill criteria for industrial-scale applications, as well as to improve cost-efficiency [40]. Biopolymer nanocomposite is a term encompassing biobased and biodegradable multiphase materials that are composed of two or more constituents in which the continuous phase is composed of the film-forming biopolymer, while the dispersed, discontinuous phase/filler phase is composed of particles with at least one nanoscale dimension [41]. Currently, the most studied biopolymer nanocomposites suitable for packaging applications are thermoplastic starch and its derivates, as well as synthetic biopolymers, such as PLA, PBS, and polyhydroxybutyrate (PHB) [39], though interest is growing regarding how to use nanotechnology to modify the more challenging natural biopolymers, e.g., chitosan, gelatin, and cellulose derivatives, for food packaging and cellulosic paper-based materials [42].

In this review, we present an overview of the nanocomposites for food packaging applications with a special focus on barrier properties, antimicrobial and antioxidant properties, migration issues, toxicity, consumer acceptability, and recyclability aspects. 

## 2. Barrier Properties of Polymer Nanocomposites

**Figure 4:**
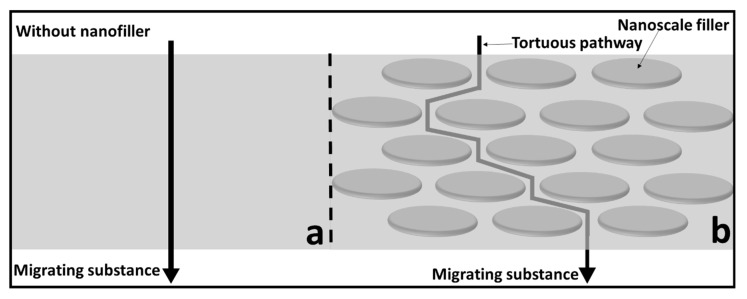
Schematic of the gas molecule diffusion through (**a**) a polymer-only barrier and (**b**) a polymer composite barrier. Uniformly dispersed nanoplates decrease the permeability by increasing the resistance through tortuosity. Adapted from [1,7].

The improvement in the barrier properties of PNCs is generally explained in the literature in terms of the increased tortuosity with the addition of fillers, as shown in Figure 4 [43]. The tortuous pathway created by the nanofillers alters the diffusion rate of the molecules, thus resulting in improved barrier properties. However, to achieve the desired improvement, the fillers must be uniformly distributed throughout the polymer matrix, which is often difficult to achieve. Another possible mechanism is the polymer–nanoparticle interaction, which can also influence the barrier properties by immobilizing the polymer strands. 

### 2.1. Polymer/Clay and Silicate Nanocomposites

Clays and other silicate materials are exceedingly stable, supposedly nontoxic in nature, and are readily available at low prices, which makes them very attractive as fillers. This is why nanoclay-based PNCs have been studied extensively for food contact applications over the last two decades. Clays, for example, montmorillonite, kaolinite, hectrite, and saponite, have been widely studied for PNC applications [44]. Typically, two main routes are used to process nanocomposites: melt compounding and in situ polymerization [45]. Fully exfoliated nanocomposite systems of polar polymers, such as polyamide with nanoclays, have been frequently reported [46,47,48]; however, in nonpolar polymer matrices, such as polyolefins, even intercalated morphologies are difficult to obtain. Two methods are generally used to improve the interaction between the hydrophobic polymer and the aluminosilicate surface of the nanoclays: the modification of clays with alkyl quaternary ammonium ions and the grafting of polyolefins with, for example, maleic anhydride used as a compatibilizer [49,50,51]. 

**Figure 5:**
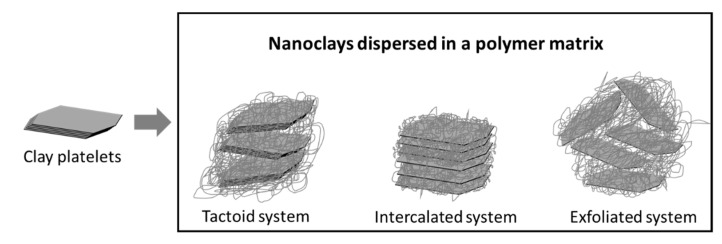
Tactoid, intercalated, and exfoliated polymer–clay systems. Adapted from [52].

The bulk properties of the PNC materials depend on the dispersion of the nanoparticles and their morphology. Monodispersed exfoliated systems show better barrier properties compared to intercalated and tactoid systems, as shown in Figure 5. 

**Figure 6:**
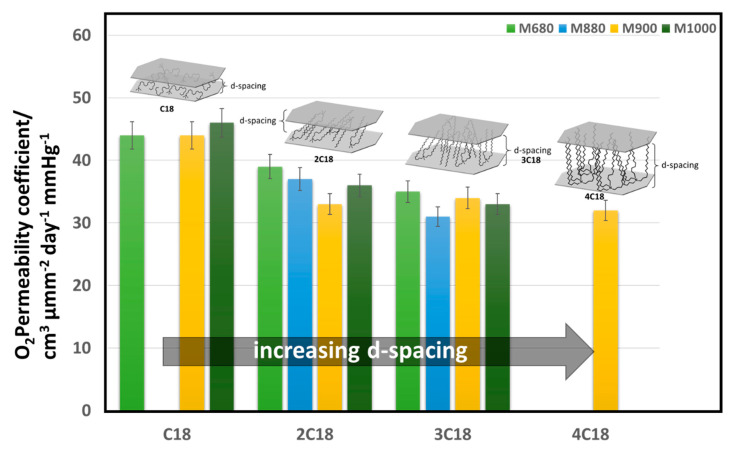
Effect of clay organic modifier on the macroscopic properties of polymer nanocomposites (PNCs). Montmorillonite with CEC/µeq g^−1^ of 680, 880, 900, and 1000 were chemically modified with trimethyl-(octadecyl)ammonium (C18), dimethyldi(octadecyl)ammonium (2C18), methyl-tri(octadecyl)ammonium (3C18), and tetra(octadecyl)ammonium (4C18) and dispersed within a linear high-density poly(ethylene) (HDPE) matrix [1,55].

Gorrasi et al. found that while tactoid and intercalated montmorillonite/polycaprolactone composite structures showed minimal improvement in water vapor barrier properties, fully exfoliated structures improve the barrier properties by almost two orders of magnitude [53]. Modifiers are also used to achieve exfoliation, for example, dispersed montmorillonite particles possess a mean interlayer distance (d-spacing) of only 3 nm, compared to the 8 nm mean interlayer separation of montmorillonite particles functionalized with octadecylamine [54]. Osman et al. have studied the effect of modifying montmorillonite clays with quaternary ammonium modifiers bearing either one, two, three, or four long alkyl (octadecyl) chains on the oxygen barrier properties, as shown in Figure 6. Larger d-spacing in the clay platelets was obtained with modifiers that have several long alkyl chains. An inverse correlation between the d-spacing and the gas permeability has been shown by the author in modified montmorillonite/polyethylene (PE) nanocomposites [55]. Clays can be incorporated in different polymers to improve their gas and water vapor barrier properties. However, the challenges related to achieving a uniform distribution of nanoclays in the polymer matrix and complete exfoliation results in a limited impact.

EVOH/montmorillonite composites have been reported with significantly improved barrier properties [56,57]. Jung et al. have reported a 32% decrease in the oxygen permeability with polypropylene (PP) incorporating clay and hollow glass microspheres [58]. Wang et al. reported a lightweight and strong microcellular injection molded PP/talc nanocomposite [59]. The tensile strength and the Gardner impact strength of the composite improved by 226% and 166%, respectively; however, the effect on the barrier properties has not been reported [59].

Jacquelot et al. reported a significant improvement in gas barrier properties (almost 100% for CO_2_ and 60% for O_2_) of a PE-based nanocomposite with oxidized paraffins as compatibilizers [45]. Another approach is in situ polymerization for obtaining polyolefin composites with nanoclays. Nikkhah et al. reported a three-fold improvement in the gas barrier properties of an in-situ-prepared PE nanocomposite compared to virgin PE [60]. Dadbin et al. proposed a single-layer PE nanocomposite film that was envisioned to replace PE multi-layer films that are commonly used in the food packaging industry [61]. Thin films prepared using blown film extrusion of the low-density polyethylene (LDPE)/linear LDPE (LLDPE)/montmorillonite organoclay nanocomposite showed a 50% decrease in oxygen permeability at only 3 parts per hundred (pph) concentration of nanoclay in the blend. Similarly, Arunvisut et al. reported a 24% decrease in oxygen permeability in an LDPE/clay nanocomposite compared to the neat LDPE. PE-grafted maleic anhydride (PEMA) was used as a compatibilizer and the clay was modified with an alkylammonium surfactant before melt mixing [62].

Numerous examples can be found in the scientific literature where nanoclays have been applied to improve the barrier properties of biobased and biodegradable materials. In a recently published study by Risyon and co-workers, the effect of incorporating different concentrations of halloysite nanotubes (HNTs) into PLA was investigated for a potential improvement in mechanical, thermal, and barrier properties. A concentration of 3 wt% HNTs was identified as the optimum, resulting in a reduction in oxygen transmission rate from 3.0 × 10^−13^ cm^3^ cm/cm^2^ s Pa for a neat PLA film to 2.0 × 10^−13^ cm^3^ cm/cm^2^ s Pa. The water vapor permeability decreased from 1.4 g m/m^2^ for neat PLA to 1.1 g m/m^2^ for PLA with 3.0 wt% HNTs. The improved barrier properties were attributed to the good dispersion of HNTs in the polymer matrix and the formation of stable hydrogen bonds between the HNT particles and the PLA, resulting in a tortuous pathway. Increased hydrogen bonding in the polymer matrix also resulted in improved mechanical properties and resistance to deformation, which was reflected in the high tensile strength and Young’s modulus (E), although there was a reduced elongation at break [63]. Nanoclay was also applied for biomaterial reinforcement in a publication from 2018, where carboxymethyl cellulose (CMC) films containing sodium montmorillonite and titanium dioxide (TiO_2_) at different concentrations were prepared using casting method and characterized. Via the addition of montmorillonite, the water vapor permeability was found to decrease by 39% and was further lowered to 50% of that of the neat CMC films by combining montmorillonite and TiO_2_. Interestingly, the study also reported a significant reduction in the moisture uptake of the films: 34% less for films with 5 wt% montmorillonite. The authors attributed the observed effect to H-bond formation between the montmorillonite and CMC, limiting the free hydroxyl groups for water absorption [64]. Other examples of montmorillonite-reinforced materials with improved barrier and mechanical properties include mucilage/montmorillonite films [65] and thermoplastic starch/montmorillonite/zinc oxide (ZnO) films [66]. Some examples of polymer nanocomposites barrier properties are listed in Table 1.

#### Layer-by-Layer Assembly

**Figure 7:**
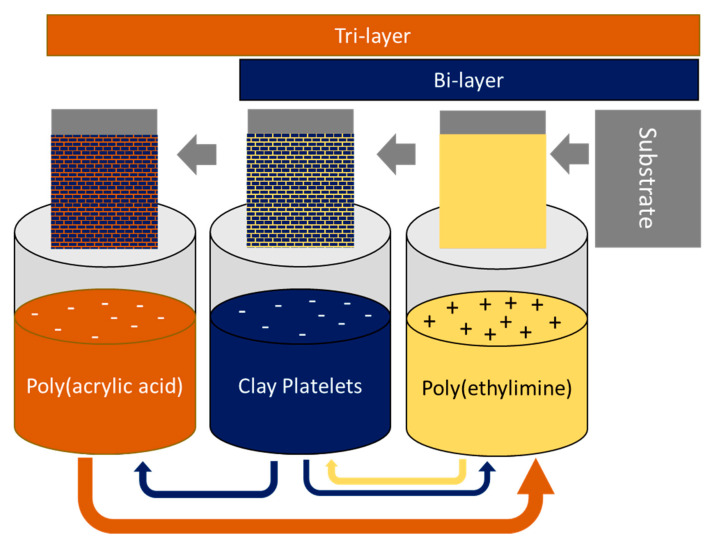
Layer-by-layer deposition process for bi- and tri-layer systems with the alternate adsorption of charged polymer and nanoclay platelets. Adapted from [99].

Jang et al. proposed a different strategy by using layer-by-layer (LBL) assembly to improve the barrier properties [97]. In their work, they developed highly structured polymer/clay films, for example, montmorillonite/poly(acrylamide) [97] and montmorillonite/poly(ethylimine) [98] structures with virtually undetectable oxygen transmission rates. Furthermore, the desired permeabilities can be obtained by adjusting the number of deposited layers and via proper selection of bi-, tri-, and quad-layer systems. The primary principle in the formation of these structures is the electrostatic interaction between the charged nanoclay platelets and the polymer surface. These LBL-assembled PNC materials are generally sensitive to moisture; however, when combined with a high moisture barrier (for example, poly(chlorotrifluoroethylene)), a very low oxygen transmission rate can be maintained, even in 95% relative humidity conditions [97]. Hagen et al. also reported on a tri-layer system consisting of cationic polyethylenimine (PEI), anionic montmorillonite, and anionic poly(acrylic acid) (PAA) deposited on a PET substrate. The deposition scheme and the resulting bi- and tri-layer structures are presented in Figure 7.

The tri-layer systems were more efficient compared to the bi-layer systems as a 10 tri-layer film had a lower oxygen transmission rate compared to a 20 bi-layer film. Furthermore, the 20 tri-layer film was at the detection limit of commercial equipment (0.005 cm^3^/m^2^ day), a 1600 times improvement over 179 μm PET (8.6 cm^3^/(m^2^ day atm)) [99]. Similarly, a quad-layer assembly of polyelectrolytes and nanoplatelets on a PET substrate was also reported [100]. In this case, the quad-layer assembly consisted of three repeating units of poly(acrylic acid) (PAA), poly(dimethyl diallyl ammonium chloride) (PDDA), and layered α-zirconium phosphate (α-ZrP). The results obtained with the quad-layer film structure are summarized in Table 2.

### 2.2. Polymer/Carbonaceous Nanocomposite

Toh et al. reported on a reduced graphene oxide (rGO)/PE nanocomposite [101]. The PNC showed a 26% improvement in the oxygen barrier property with the addition of 0.3 wt% rGO, where a further increase in the amount of rGO from 0.3 wt% did not lead to any significant changes in the oxygen permeability. Furthermore, an improvement of 45% in the stiffness was also reported for the PNC [101]. Kim et al. coated an EVOH/graphene oxide composite on the surface of a biaxially oriented (BO) PP film. The oxygen transfer rate decreased from 1189 (bare BOPP) to 0.6 cc m^−2^ day^−1^ atm^−1^ (coated BOPP) [102]. There are several reports of PLA-carbon nanotube composites with improved thermal stability and better mechanical properties [103,104]. 

### 2.3. Polymer/Silica Nanocomposites

Khankrua et al. reported on PLA, PBS, and poly(3-hydroxybutyrate-co-3-hydroxyvalerate) (PHBV) nanocomposites with fumed silica that were prepared using twin-screw extrusion [105]. At low silica loadings (0.1–0.5 wt%) there was a minor improvement in the mechanical properties; however, at higher loadings, the mechanical properties deteriorated due to agglomeration of the particles. Surface-modified nanoparticles have also been reported in the literature to achieve a uniform distribution in the polymer matrix. PE glycol methyl ether (PEGME)-grafted silica particles were described by Lai et al. [106]. The grafting via amino silane altered the surface properties and resulted in better dispersion of the modified silica particles in the polymer matrix with improved tensile strength compared to the unmodified particles. Similarly, a PLA composite with nanosilica grafted with lactic acid and oleic acid was reported by Yan et al. and Zhu et al.; significant improvement in the elongation at break and the flexibility of the PNC was achieved [107,108]. Yuan reported on a PP nanocomposite with PP grafted silica nanoparticles. The resultant PNC had a relatively uniform distribution of the nanoparticles in the polymer matrix and exhibited improved melt strength and thermal stability [109]. 

Even though the addition of silica nanoparticles can improve the mechanical and thermal properties of polymer matrices, the enhancement of gas barrier properties is not as straightforward to achieve. It has been reported that the gas barrier properties can be greatly improved through vapor deposition of thin silica and alumina films on polymer substrates; however, these films are prone to cracking upon bending [110,111]. Batra et al. studied gelatin/chitosan/silica biopolymer nanocomposites and they documented a significant reduction in water vapor permeability from 25.21 g mm/kPa m^2^ day for the neat gelatin films to 3.30 g mm/kPa m^2^ day after the incorporation of 10 wt% silica nanoparticles. However, they also found that the inclusion of nanoparticles increased the moisture content of the films. This was discussed as either being due to an increased entrapment of water in the polymer matrix, the binding of water to chitosan nanoparticles, or increased availability of hydroxyl groups as a spacing between the gelatin chains increased [112].

There are several reports on the incorporation of silica nanoparticles into PP matrices, with various effects on the barrier properties. Bracho et al. studied how the size and surface modification of silica nanoparticles affect the water vapor permeability of PP nanocomposites [113]. The results showed increased permeability to water, which was explained in terms of the hygroscopic nature of the silica surface. The effect was also size-dependent, with a smaller size (higher surface area) giving higher water vapor permeability values. Surface modification of the nanoparticles did further increase the permeability, possibly due to the formation of agglomerates, which subsequently created void channels in the polymer, and thus increased the permeability for water vapor. Dougnac et al. also studied the size effect of silica nanoparticles on the permeability of oxygen, nitrogen, and water vapor through a PP–silica nanocomposite [114]. The permeability of oxygen and nitrogen increased in almost all the nanocomposites with the increase in the nanoparticle diameter. The formation of void channels, where the molecules can travel freely, were also observed here. Only the smallest particles (12 nm in diameter) reduced the permeability of oxygen and nitrogen. It was concluded that these small particles form aggregates that function as larger barriers, and thus decrease the permeability through tortuous pathway mechanisms. The water vapor transmission rate also increased for all particles, except for the smallest ones. However, this increase was inversely proportional to the nanoparticle diameter, which was also explained in terms of the hygroscopic nature of the silica surface adsorbing the water vapor, thus increasing its solubility in the nanocomposite matrix.

In contrast, Vladimirov et al. prepared PP–silica nanocomposites from fumed silica and observed that the permeability rates of oxygen and nitrogen were reduced with increasing silica content [115]. This was explained in terms of the tortuous pathway. A higher concentration of fumed silica led to larger agglomerates, which in this study resulted in better barrier properties. However, it can be noted that the size of the individual silica nanoparticles comprising the agglomerates was 12–15 nm, which is in good agreement with the study discussed above, where only the 12 nm particles improved the gas barrier properties. Another study on PP–silica nanocomposites, by Vassiliou et al., confirmed these findings [116]. Here, the introduction of fumed silica into a PP matrix resulted in a significant reduction of the oxygen, nitrogen, and carbon dioxide permeability rates with increasing silica concentration. The highest reduction was observed at 10 wt% silica content.

These results illustrate the importance of the right size, morphology, and surface chemistry of the nanofillers. The choice of compatibilizer is also critical for improving the compatibility between the polymer and the nanofillers, and thus reducing the risk of the formation of void channels. Additionally, the choice of method for creating the nanocomposites, as well as the optimization of the parameters within, i.e., extrusion parameters, also have a significant impact. 

### 2.4. Other Nanofillers

Nanoparticles other than silica have also been tested, for example, a magnesium oxide (MgO)/PLA composite has been reported by Swaroop et al., with 25% improved gas barrier properties [117]. Similarly, calcium carbonate composite with PP has been shown by Chan et al., with significantly improved mechanical properties [118]. Metal oxides, such as TiO_2_ and ZnO, were found to be potent nanofillers for the reinforcement of biodegradable materials. Alizadeh-Sani et al. developed whey protein isolate (WPI) films that were modified via the use of different concentrations of cellulose nanofibers, TiO_2_, and rosemary essential oil. The characterization of the films revealed a significant increase in water resistance after the addition of TiO_2_ and essential oil. The addition of TiO_2_ nanoparticles and essential oil also significantly reduced the moisture absorption in the films, where SEM microscopy confirmed the homogeneous dispersion of the TiO_2_ nanoparticles. With regard to mechanical properties, the addition of cellulose nanofibers (7.5 wt%) and TiO_2_ (1 wt%) resulted in a significant increase in tensile strength (TS) and Young’s modulus, while the elongation at break was reduced after the inclusion of nanoparticles, a finding that was supported by similar studies [119].

An interesting and widely studied approach for reinforcing biomaterials is via the use of bio-nanofillers, which are biodegradable in the same manner as the matrix biopolymers. Material combinations in recently published studies include konjac glucomannan/chitin nanocrystals [120], κ-carrageenan/cellulose nanocrystals [121], gelatin/nanocellulose, and starch/nanocellulose [122]. Yadav and Chiu compared the mechanical properties and water vapor permeabilities of neat κ-carrageenan films to those of κ-carrageenan biopolymer nanocomposite films reinforced with cellulose nanocrystals. The hydrophobicity of the materials significantly increased, which was reflected in a water contact angle increase from 23.30° for neat films to 57.75° for κ-carrageenan films loaded with 7 wt% cellulose nanocrystals. This finding was substantiated by a decrease in water vapor permeability from 8.93 gm^−1^ s^−1^ Pa^−1^ for the neat κ-carrageenan films to 4.69 × 10^−11^ gm^−1^ s^−1^ Pa^−1^ for the modified films [121].

## 3. Active Nanocomposite Films

Active materials are defined in the European Commission Regulation (EC) No. 450/2009 as follows: “active materials and articles means materials and articles that are intended to extend the shelf-life or to maintain or improve the condition of packaged food; they are designed to deliberately incorporate components that would release or absorb substances into or from the packaged food or the environment surrounding the food.” In addition, conditions for active materials and articles are given in Framework Regulation (EC) 1935/2004. 

Nanocomposites with antimicrobial and antioxidant properties have the potential to prolong the shelf life of the food products by suppressing their enzymatic, oxidative, and microbial spoilage. Metal and metal oxide nanoparticles, such as silver, titanium, copper, zinc, ZnO, MgO, and TiO_2_, have been reported in the literature for antimicrobial packaging applications [122,123,124,125,126,127]. The addition of antioxidants may hinder the oxidation of, e.g., fat and proteins.The antioxidants decelerate the development of off-flavor and improve the color stability of the food. The oxidation of food products may be reduced by removing or limiting the presence of oxygen, e.g., by using high barrier packaging materials and an anaerobic atmosphere, in addition to active packaging via the use of an oxygen scavenger [128]. Moreover, the prevention of lipid oxidation may be obtained using antioxidant-releasing packaging systems and synthetic antioxidants (e.g., butylated hydroxy-toluene (BHT)), which have been widely used in food packaging. However, the use of natural antioxidant compounds has gained increased interest in recent years. Tocopherols, polyphenols, and plant extracts, such as essential oils, have been reported to increase the oxidative stability of different food products [128].

### 3.1. Novel Encapsulation Techniques

Extensive research efforts are being put into the development of active packaging solutions based on natural antimicrobial and antioxidant compounds [129,130,131], including encapsulation strategies to stabilize these compounds in the food packaging material [17,132,133]. Regarding encapsulation, different organic systems have been utilized, such as emulsions. Oil-in-water Pickering emulsions that were stabilized by cellulose nanofibrils were exploited in a study by Valencia et al. to prepare thin films [134]. Encapsulation of the lipophilic compound curcumin in these composite films provided antimicrobial activity against *Escherichia coli*, as well as antioxidant properties. The radical scavenging activity increased as a function of the curcumin amount, and the authors reported up to 30% radical scavenging activity of the films.

Recently, inorganic nanostructures, such as nanoclays, have been demonstrated to be suitable carriers for lipophilic and/or volatile natural compounds, such as essential oils and their active components. Shemesh et al. utilized HNTs for encapsulating carvacrol (used as a model essential oil), and further incorporated these HNTs/carvacrol hybrids into LDPE films via melt-compounding [135]. It was shown that HNTs greatly improve the thermal stability of the volatile carvacrol molecules, and thus function as carriers to allow the volatile compound to be incorporated into polymers via a high-temperature melt-compounding process. It was further demonstrated that carvacrol retained its antimicrobial functionality after the high-temperature processing, and the encapsulation slowed down the diffusion rate of carvacrol from the films compared to LDPE films that incorporate carvacrol without HNT carriers. These film properties had a significant influence on the shelf life, exhibiting bactericidal efficiency against *E. coli* for up to six weeks. Additionally, the films showed good antibiofilm properties against *Listeria innocua*, as well as antifungal activity against *Alternaria alternata.* The authors also demonstrated the performance of the films in inhibiting fungi growth on bread and an antibacterial effect in soft cheese. High-temperature melt-compounding was further used by the same authors [136] to produce PP films that incorporated HNTs loaded with carvacrol. The HNTs were well-dispersed in the PP matrix, and these nanocarriers decreased the diffusivity of carvacrol by almost 30% in comparison to films where carvacrol was directly incorporated into PP films. A high antibacterial and antifungal activity of the films were demonstrated on *E. coli* and *A. alternata*; however, no difference in the antimicrobial effect could be seen between the PP/carvacrol and PP/(HNTs–carvacrol) films. Molecular dynamics simulations performed in the study revealed a strong interaction between the PP polymer and carvacrol, which had a significant role in retaining the volatile compound in the PP matrix. Even though the encapsulation of carvacrol did not affect the direct antimicrobial activity, the HNTs were found to positively affect the structure of the film by resulting in a higher crystalline order level. In addition, the HNTs improved the mechanical properties of the film, as well as delayed the carvacrol release. Another approach for incorporating HNTs loaded with an active compound into food packaging films is through coating techniques. Tas et al. used the layer-by-layer assembly technique to coat PE film with thin layers of chitosan and carvacrol-loaded HNTs [137]. The antimicrobial activity of the films was evaluated against the food pathogen *Aeromonas hydrophila* and resulted in an 85% decrease in the viability of the bacteria due to the release of carvacrol molecules. The films also reduced the aerobic count on chicken meat surfaces by 48% compared to neat PE films. However, the oxygen barrier properties were not compared for the coated and neat PE films. 

Another inorganic nanomaterial that has been utilized to improve the incorporation of volatile active compounds into polymer films is non-porous silica nanoparticles. Sepulveda et al. incorporated cinnamaldehyde into nanocomposite films based on PLA and silica nanoparticles [138]. The silica nanoparticles were surface-functionalized with lactic acid to improve the dispersibility, as well as the compatibility with the PLA matrix. Cinnamaldehyde was incorporated by supercritical impregnation and was reported to be physically entrapped between the silica nanoparticles. Due to the affinity between the cinnamaldehyde and the nanoparticles, the diffusion rate of cinnamaldehyde was decreased by 74%. The surface-functionalized nanoparticles were further shown to decrease the oxygen permeability by 44% compared to the pure PLA films. Ellahi et al. also concluded that silica nanoparticles can sustain the release of *Pistacia atlantica* gum essential oil from a PP film [139]. The PP polymer was coated with silica nanoparticles and the essential oil. The film was reported to maintain its antibacterial properties for up to 35 days and was investigated to be used for preserving milk.

Furthermore, porous silica nanoparticles, which are well-established carriers for active compounds in the biomedical field, have also lately emerged as carriers in the field of food packaging applications. Wu et al. studied biopolymer nanocomposite chitosan films that were incorporated with curcumin-loaded mesoporous silica nanoparticles [140]. These films exhibited a pH-responsive and sustained release of curcumin, which resulted in antimicrobial activity against *Staphylococcus aureus* and *E. coli*. Porous silica materials have also been shown to be suitable candidates for encapsulating volatile compounds. Ruiz-Rico et al. compared the antimicrobial activity of four different volatile compounds (carvacrol, eugenol, thymol, and vanillin) by covalently attaching them to the surface of three different silica supports: fumed silica, amorphous silica, and MCM-41 (a mesoporous material with a high specific surface area and pore volume) [141]. Due to the anchoring of the active compounds, the antimicrobial effect here was based upon direct contact between the silica supports and the bacteria, and not by the release of the compounds. The immobilization of the active compounds greatly enhanced the antimicrobial activity against *L. innocua* and *E. coli*. However, the Gram-positive bacteria (*L. innocua*) was more sensitive to the active compounds, as is generally established. In addition, the different combinations of silica support and active compounds yielded distinct antimicrobial activity. Furthermore, sensory evaluations revealed a significant reduction of the aroma intensity of the immobilized thymol, which is studied in pasteurized skimmed milk. In another study, by Melendez-Rodriguez et al., MCM-41 silica nanoparticles were utilized as carriers for eugenol to be further incorporated into PHBV films via electrospinning [142]. Here, eugenol was physically adsorbed to the silica support to allow for a sustained release. The biopolymer films showed antimicrobial activity against *S. aureus* and *E. coli*, which increased after 15 days due to the volatile portion that had accumulated in the headspace of the closed system.

### 3.2. Bio-Based and Biodegradable Nanocomposite Films

Research on active biopolymer nanocomposite materials is an emerging field that, if successful, may compensate for some of the shortcomings of these bio-based and biodegradable materials in terms of, e.g., oxygen barrier properties. The use of antimicrobial or antioxidative components in combination with nanofillers creates an additional hurdle for spoilage bacteria or oxidative spoilage and may thereby narrow the gap with the performance of conventional plastic packaging materials in terms of the obtained shelf-life of a packaged product.

Montmorillonite has been applied in combination with different essential oils in biopolymers, e.g., chitosan/montmorillonite/ginger essential oil [143], chitosan/montmorillonite/rosemary essential oil [144], and soy protein/montmorillonite/clove essential oil [145]. Pires and co-workers studied chitosan films that were reinforced with sodium montmorillonite and incorporated with rosemary or ginger essential oil. The films were applied in packaging trials with fresh poultry meat. The authors concluded that montmorillonite incorporation alone resulted in reduced lipid oxidation (thiobarbituric acid reactive substances (TBARS)) of the meat, which was attributed to an increased barrier against UV light, as well as an assumed increased oxygen barrier (not measured). The incorporation of essential oils, in addition to montmorillonite, only reduced the lipid oxidation and did not inhibit the microbial growth on the meat [146]. 

Metal oxides have also been studied for their antimicrobial properties regarding the enhancement of biodegradable materials, e.g., CMC/mucilage/ZnO [147] and chitosan/cellulose acetate phthalate/ZnO biocomposites [148]. However, in many studies, an additional antimicrobial effect was targeted by combining metal oxides with essential oils. Examples of such studies from the last few years involve alginate/ZnO/essential oil [149], WPI/cellulose nanofiber/TiO_2_/rosemary essential oil [150], and gelatin/ZnO nanorods/clove essential oil biopolymer nanocomposites [151]. 

In a study from 2016, a chitosan/TiO_2_ nanocomposite coating was developed and tested for its performance on cellulosic paper. The biopolymer nanocomposite coating was found to improve the mechanical properties of the paper packaging material, as well as inhibit microbial growth on the material’s surface [152]. Biodegradable coatings with chitosan incorporating vermiculite nanoclay on a PET substrate, resulting in a significant decrease in the oxygen permeability, have also been reported [153].

One of the hurdles of the industrial use of biodegradable materials is their higher cost compared to conventional plastic materials. The use of nanotechnology as a reinforcement for these materials may reduce the production cost, as lower amounts of biodegradable polymers are required to obtain the desired properties [40].

The effect of nanofillers on the biodegradability of the biopolymer nanocomposite materials and how to tailor these materials for biodegradability are emerging questions that calling for research-based answers. Studies have demonstrated that some nanofillers alter the biodegradability and the rate of microbial degradation of biopolymers due to changes in their crystallinity. Biodegradation may be positively or negatively affected based on the net chemical interaction between the biopolymer matrix and the nanofiller [154,155].

Mishra et al. argued that the key challenges to address in order to reach commercialization for biopolymer nanocomposite materials is developing an effective separation route for the extraction of nanofillers from the biopolymer after use, achieving compatibility between nanofillers and biopolymer matrix, and finding suitable techniques for the processing of biopolymer nanocomposite materials [155]. Souza and Fernando pointed to the risk of environmental contamination and potential ecotoxicity as nanoparticles are released from the biomaterials during degradation. Research is required to clarify the fate of nanoparticles when released into the environment and whether they have the potential to bioaccumulate in the food chain or act as contaminants [154].

Some examples of the antimicrobial properties of the polymer nanocomposites are listed in Table 3.

## 4. Migration

**Figure 8:**
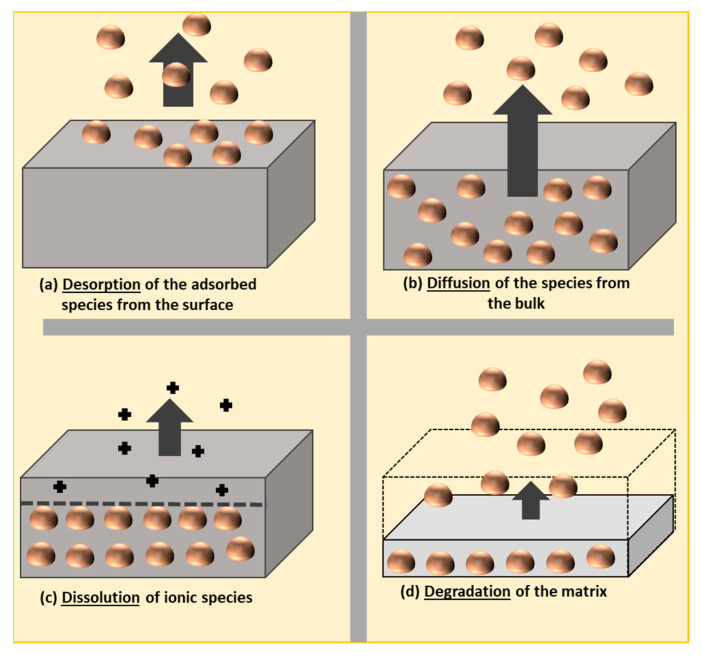
Potential release pathways, known as “the 4Ds”: desorption, diffusion, dissolution, and degradation of engineered nanomaterials from polymer nanocomposites, as proposed by Noonan et al. [171].

Generally, the release of nanomaterials can take place through desorption, diffusion, dissolution, and degradation phenomena, as explained by Noonan et al. [170] (Figure 8). According to the European legislation on plastic materials, “the most comprehensive specific EU measure is Regulation (EU) No 10/2011 on plastic materials and articles. It sets out rules on the composition of plastic food contact materials (FCMs) and establishes a Union List of substances that are permitted for use in the manufacture of plastic FCMs. The Regulation also specifies restrictions on the use of some substances and sets out rules to determine the compliance of plastic materials and articles” [170].

According to this regulation, the overall migration limit is 10 mg/dm^2^, which translates to 60 mg/kg food (considering cubic packaging with 1 kg of food). A maximum limit of 0.01 mg/kg in food is proposed for the migration of a non-authorized substance through a functional barrier. Substances that are mutagenic, carcinogenic, or toxic to reproduction are prohibited for use. The specific migration limits for the constituents of different plastic materials and articles are provided in the union list of substances; however, for the substances for which no specific migration limit or other restrictions are provided, a generic specific migration limit of 60 mg/kg food is applied. According to the EU Regulation No. 10/2011, nanoparticles should be assessed on a case-by-case basis. Standardized test conditions, including testing time, temperature, and test medium (food simulant) are stated in the regulation [172]. 

Using nanotechnology for the modification of food packaging materials is still a novel field, which is one of the reasons for the limited number of migration studies of polymer nanocomposites in the literature [173]. Furthermore, the limited available methods for qualitative and quantitative analysis and difficulties in characterizing nanoparticles in composites makes it harder to perform such studies [174]. Avella et al. have studied the migration of montmorillonite from biodegradable starch/clay nanocomposite. They reported that the overall migration of montmorillonite was within the acceptable range of 60 mg/kg [175]. There are contradictions in the literature with regard to the migration of nanoparticles, with some authors indicating that the diffusion of nanoparticles is possible; however, this is not the consensus [154]. 

Stromer et al. recently critically reviewed the migration potential of nanoparticles in food contact plastics. Among various migration studies, they also looked at the diffusion models explaining the migration of nanoparticles [176]. They concluded that only very small nanoparticles (1–2 nm in diameter), which are in the size range of larger plastic additives, may potentially migrate. Larger nanoparticles (from 5 nm in diameter) are not likely to migrate, even at high concentrations, if these are immobilized in the polymer. This can, however, not be experimentally verified since the expected concentrations are below the detection limit of any analytical technique. Thus, it is important that manufacturers ensure that nanoparticles are always fully incorporated into the polymer matrix, to not have any possibility to migrate into food. One possibility to avoid the potential migration of nanofillers is to use multilayer structures. Garafalo et al. reported on PA/PE multilayer nanocomposite films [177]. Overall migration studies showed that this solution is feasible and can be used to overcome the problem of possible nanoparticle migration.

## 5. Toxicity and Safety Aspect

Currently, according to the “Plastic Food Contact Materials” Regulation (EU) 10/2011, only nanoparticles authorized and specifically mentioned in the specification of Annex I of the regulation can be used in plastic packaging for food. This also applies to nanoparticles that are intended to be used behinda functional barrier. 

Nanoparticles, which were initially listed in the specifications, and thus authorized, are silica, carbon black, and titanium nitride [172,178]. Titanium nitride nanoparticles are typically used as an additive in PET bottles, for which a concentration of up to 20 mg/kg is allowed. However, the specific migration limit (SML) and the total specific migration limit (SML (T)) are not specified in the regulation. Similarly, carbon black nanoparticles are used as an additive with a maximum use level of 2.5 wt% without specifications for the SML or SML (T) in the regulation. Silica nanoparticles are also used as an additive with no SML or SML (T) being specified [170,172,179].

For food contact plastic packaging containing particles/fillers other than the three listed above, an application must be submitted to European Food Safety Authority (EFSA), which contains specific information regarding the migration, toxicology, and possible exposure for their authorization.

The basis of the safety evaluation of the nanomaterials in food packaging is to determine the “exposure risk” toward nanomaterials. The nanomaterials can potentially migrate from a food contact material (FCM) to the packed food, either through diffusion or dissolution. Migration tests are usually the first to be performed in the safety evaluation. If the migration tests show no measurable migration of nanomaterials, no human exposure will likely occur. Consequently, there will be no overall risk to the consumer and further toxicity testing will not be needed [176]. In the case where it is demonstrated that nanomaterials can migrate and enter the food matrix, the solubility of the nanomaterial in the food matrix and/or upon gastrointestinal passage is of relevance [178]. For nanomaterials that completely dissolve in the food matrix, the hazard and risk upon exposure will be similar to its bulk form [178]. Furthermore, it is also very important to distinguish between the release of a particle and the migration of dissolved ions. For example, it has been reported in the literature that silver ions are significantly more toxic compared to silver nanoparticles [180]. 

For nanomaterial detection and characterization, only a few techniques exist that are sensitive and selective enough. For nanomaterial analysis within a polymer matrix, X-ray diffraction (XRD), X-ray fluorescence (XRF), and high-resolution imaging techniques, such as transmission electron microscopy (TEM), are most commonly used. For nanomaterial detection in food simulants, asymmetric flow field-flow fractionation (AF4) coupled with a multi-angle light scattering (MALS) or dynamic light scattering (DLS) detector is used, which allows for the separation of nanomaterials according to their size, which allows for a precise measurement of the particle size distribution [181,182]. For chemical analysis, AF4 is coupled with element-specific techniques, such as inductively coupled plasma-mass spectrometry (ICP-MS) [183]. Basic ICP-MS does not differentiate between nanosized elemental metal and metal ions [184]. Single-particle (sp)-ICP-MS is a new technique that can quantitatively distinguish between dissolved and particulate species and works well if appropriate dispersants and standard nanomaterials with defined particle sizes are used [185]. However, in practice, sp-ICP-MS measurements are often of limited use due to difficulties when measuring complex samples, such as dispersions of nanomaterials with broad particle size distributions, or incompatible matrices, e.g., organic solvents [186]. 

Among the three different routes, namely, dermal contact, inhalation, and oral ingestion, the oral uptake of nanoparticles from food or through migration from the packaging can be a significant exposure source [154]. Different model systems have been used to study the toxicity of the nanoparticles, both in in vitro and in vivo experiments. Typically, human and rodent cell lines, especially from the intestine, liver, lung, and skin, are utilized for in vitro experiments, while rodents are mostly used for in vivo methods [187]. The toxicity testing of nanomaterials usually starts with determining the absorption, distribution, metabolism, and excretion (ADME) to identify the kinetics and possible accumulation of nanomaterials in the body. In cases where systemic exposure can be excluded, EFSA requires at least in vitro genotoxicity and in vivo local effects testing to be carried out. Where absorption of nanomaterials upon oral exposure has been demonstrated, hazard identification of the nanomaterial by appropriate in vitro and/or in vivo studies for mutagenicity and repeated dose toxicity (for 90 days) is required. In vivo genotoxicity testing is required when initial in vitro tests show positive results or when the results return inconclusive [178].

The nanoparticle exposure risk during the manufacturing and processing stages can be minimized through proper management of the risks, as detailed in the ISO/TS 12,901 series [188,189]. There is a minimal risk of exposure during the post-production transportation of nanomaterial and nanomaterial-polymer resins since the transportation is mainly carried out in sealed containers.

Maisanaba et al. evaluated the cytotoxicity and mutagenicity of clays and organo-modified clays. The authors showed cytotoxicity and genotoxic damage induced by organo-modified clay (tested concentration range 0–250 µg/mL); however, unmodified clay mineral did not show any toxicity in the concentration range of 0–125 µg/mL [190]. Most of the in vitro toxicity reports on clay minerals showed cell death; in contrast, human and animal data exhibited very low toxicity. Furthermore, different parameters, including (i) the exposure conditions, such as the concentrations or exposure times assayed; (ii) the experimental models selected; (iii) the modifiers or surfactants incorporated in their structures and their concentrations; (iv) the sensitivity of the assays performed are used to express the toxicity profile of clay minerals and the derived nanocomposites. In this regard, a case-by-case toxicological evaluation is always required [187]. 

Silica or silicon dioxide nanoparticles are used as a food additive (E551). Guo et al. studied the toxicity of silica particles in in vitro model consisting of Caco-2 and HT9-MTX co-cultures [191]. They reported that the exposure to silica nanoparticles altered the functionality of intestinal epithelial cells. On the other hand, the European Food Safety Authority has also published a re-evaluation report of silica as a food additive. The panel concluded that, “based on the available database, there was no indication for toxicity of E 551 at the reported uses and use levels” [192]. In general, the toxicity of the nanoparticles is greatly influenced by their size, dispersion, concentration, morphology, etc. In this respect, an individual toxicological evaluation is always vital.

## 6. Consumer Acceptability

Nanotechnology can potentially be applied to a broad range of food and food packaging applications. It can be utilized either to develop functional and novel foods where nutrients are encapsulated and delivered in food or to improve functional properties (for example, barrier, mechanical, thermal, sensing, and antimicrobial properties) of the packaging materials. Consumer studies performed on the topic of nanotechnology for food packaging have, however, mainly focused on antimicrobial packaging (silver-based) [3,193]. Since the antimicrobial activity of the silver-based materials is due to the release of silver ions, it is often a cause of concern among consumers, with questions regarding safety and the long-term effects on human health [193].

Siegrist et al. studied the public acceptance of nanotechnology for foods and food packaging [3]. Their results support the hypothesis that nano-inside (e.g., foods) is perceived as less acceptable than nano-outside (e.g., packaging). Later, Siegrist et al. performed a more elaborative study on the perceived risks and benefits of nanotechnology for food packaging [4]. Among other applications, they also included decay-inhibiting films, antibacterial food containers, oxygen-absorbing films, oxygen-blocking plastic bottles, stronger packaging films, UV-protection packaging, barcodes for guaranteed food security, etc. in their study to cover a broad range [4]. They concluded that the use of nanotechnology for packaging is considered less problematic in the public view and that consumers may be more likely to accept innovations related to packaging than those related to foods. However, the consumer studies cited here are more than seven years old and consumer awareness on the topic of nanotechnology and food packaging might have transformed during the last seven years. 

## 7. Recyclability of Nanoreinforced Plastic Packaging

Another important aspect to consider is the recyclability of the polymer nanocomposite materials. Mechanical recycling of the plastic composite materials has been reported in the literature [194,195]; however, more research is needed in this area. Sánchez et al. have performed a systematic assessment of the recyclability of nanoreinforced plastic packaging [196]. The evaluation was carried out through recycling tests, where conventional recycled packaging films without nanomaterials were subjected to extrusion processing in combination with increasing concentrations of nanoreinforced plastic films. Only one recycling cycle was considered. The evaluation was focused on PE, PP, and PET monolayer films used in the food sector that were reinforced with 4 wt% of four nanoparticles (ZnO, Ag, nanoclay, and CaCO_3_). Specifically, six plastic film materials were included in the study: PE + CaCO_3_, PE + nanoclay, PP + Ag, PET + ZnO, PET + Ag, and PET + nanoclay.

The authors concluded that for PE and PP, the introduction of nanomaterial does not greatly affect the final recycled material regarding mechanical properties and material quality (in terms of haze, smells, pinholes, etc.) compared to conventional recycled plastic. Regarding PET, results show that the increasing addition of nanomaterial into the recycled PET matrix (especially PET–Ag) could influence important properties of the recycled material, due to a slight degradation of the polymer, such as increasing pinholes, degradation fumes, and elongation at break [194].

## 8. Conclusions

Nanotechnology applied in the form of nanocomposites has shown great promise for food packaging applications. In line with the increased focus on sustainability, materials from renewable resources are increasingly demanded and nanotechnology as reinforcement can contribute toward overcoming some of the hurdles for the industrial use of these biomaterials.

The barrier properties of the polymers can be improved significantly by utilizing nanofillers if their uniform distribution is achieved. Nanoclays are mostly reported in the literature for improved barrier properties of the polymer composites in traditional fossil-based polymers, as well as bio-based and biodegradable materials. Surface grafting approaches and layer-by-layer assembly methods have been described as being able to overcome the challenges of agglomeration and to achieve a uniform distribution of nanofillers. 

Silica nanoparticles can improve the mechanical and thermal properties of polymer matrices, in addition to the enhancement of gas barrier properties. However, the physicochemical properties of the nanofillers, such as the size, morphology, and surface chemistry, are of importance and have to be optimized with regard to the specific application. Nanocomposites with antimicrobial and antioxidant properties also have the potential to improve preservation and prolong the shelf life of food products. Nanoclays and recently also silica particles have been reported as encapsulation matrices for natural active compounds, such as essential oils and their major components. Encapsulation protects the volatile active compounds during high-temperature processing and may allow for a prolonged release. Furthermore, the negative organoleptic effects of essential oils and their active components on food products can also be minimized through encapsulation.

There is a knowledge gap in the area of the migration, toxicity, consumer acceptance, and recyclability of nanoreinforced plastic packaging. The application of nanotechnology for food packaging applications still has a number of important issues that are yet to be resolved, mainly regarding safety concerns (migration), the industrial scale-up, and recyclability. In the European Union, there are strict regulations in place for food contact materials. Currently, according to the “Plastic Food Contact Materials” Regulation (EU) 10/2011, only nanoparticles from silica, carbon black, and titanium nitride are authorized. For food contact plastic packaging containing particles/fillers from other materials, an application must be submitted to the EFSA containing specific information regarding migration, toxicology, and possible exposure for their authorization. In relation to sustainability and the circular economy, waste management and recyclability of the polymer nanocomposite materials is of importance and needs to be considered. Mechanical recycling of the plastic composite materials has been reported; however, the information is limited, thus more research is needed.

## Figures and Tables

**Table 1:** Examples of polymer nanocomposites and the changes in their barrier properties expressed as an improved ratio in permeability, summarized from [1,7,8,9,10,11]. ^a^ Organo-modified montmorillonite, ^b^ graphene oxide nanosheet, ^c^ dodecyl amine-modified graphene, ^d^ functionalized graphene oxide, ^e^ thermally reduced graphene, ^f^ graphite nanoplatelets, ^g^ reduced graphene oxide.

Polymers	Abbreviations	Filler Type	wt%	P(O_2_) as Improved Ratio	P(H_2_O) as Improved Ratio	Ref.
Poly(imide)	PI	OM-MMT ^a^	8	13	7.4	[67]
Poly(imide)	PI	OM-MMT	2	19.8		[68]
Poly(styrene)	PS	OM-MMT	16.7	2.8		[69]
Poly(amide)	PA	OM-MMT	5.5	>1100		[70]
Poly(ethylene terephthalate)	PET	Na-MMT	5	15.6	1.2	[71]
Poly(ethylene terephthalate)	PET	MMT	5	2.23	1.15	[72]
Ethylene-vinyl alcohol	EVOH	Kaolinite	5	3.0–4.0	1.2	[73]
Poly(lactic acid)	PLA	MMT	5	1.16	1.21	[72]
Poly(lactic acid)	PLA	OM-MMT	5	1.2–1.9	1.7–2.0	[74]
Poly(lactic acid)	PLA	Synthetic mica	4	2.8		[75]
Poly(vinyl chloride)	PVC	SiO_2_	3	1.6	2.8	[76]
Poly(propylene)	PP	CaCO_3_	3	1.4		[77]
High-density poly(ethylene)	HDPE	OM-MMT	4	1.2–1.7		[55]
High-density poly(ethylene)	HDPE	OM-MMT	5	2.8–2.9	1.8–2.4	[78]
Low-density poly(ethylene)	LDPE	OM-MMT	4.76	2.2		[61]
Poly(styrene)	PS	MMT	6		3.33	[79]
Poly(styrene)	PS	MMT	10		2.16	[80]
Linear low-density poly(ethylene)	LLDPE	Cloisite 25A	5		4.76	[81]
Poly(imide)	PI	MMT	8		5.88	[67]
Poly(imide)	PI	Synthetic mica	2		10	[44]
Poly(lactic acid)	PLA	MMT	5		2	[74]
Poly(lactic acid)	PLA	MMT	10		12.5	[82]
Poly(styrene)	PS	Graphene	2.27	2.56		[83]
Poly(lactic acid)	PLA	GONS ^b^	1.37	1.8		[84]
Linear low-density poly(ethylene)	LLDPE	DA-G ^c^	1	1.88		[85]
Poly(ethylene terephthalate)	PET	fGO ^d^	3	41.6		[86]
Ethylene-vinyl alcohol	EVOH	TRG ^e^	0.5	5000		[87]
Poly(ethylene terephthalate)	PET	GNPs ^f^	1.5	100		[88]
Poly(imide)	PI	rGO ^g^	30	14.3		[89]
Poly(ethylene terephthalate)	PET	OM-MMT	5	2.2		[90]
Poly(ethylene terephthalate)	PET	OM-MMT	5	3.2		[91]
Poly(ethylene terephthalate)	PET	OM-MMT	1	1.81		[92]
Poly(styrene)	PS	OM-MMT	2	2.9		[93]
Poly(propylene)	PP	OM-MMT	4	1.85		[94]
Poly(propylene)	PP	OM-MMT	7.5	2.27		[95]
Linear low-density poly(ethylene)	LDPE	OM-MMT	7	1.31		[62]
Linear low-density poly(ethylene)	LDPE	OM-MMT	0.5	4		[96]

**Table 2:** Barrier properties of PET and PET–quad-layer systems [100]. OTR: oxygen transmission rate, OP: oxygen permeability, WVTR: water vapor transmission rate, WVP: water vapor permeability.

Sample	OTR (cm^3^/(m^2^ 24 h))	OP (cm^3^ mm/(m^2^ 24 h))	WVTR (g/(m^2^ 24 h))	WVP (g mm/(m^2^ 24 h))
PET	57.0	1.43	9.97	0.25
PET-(QL)1	31.3	0.78	9.30	0.23
PET/(QL)3	12.8	0.32	9.22	0.23
PET-(QL)19	0.87	0.02	8.50	0.20
PET-(QL)20	Undetectable	-	8.45	0.19

**Table 3:** Examples of antimicrobial properties of the polymer nanocomposites.

Active Component	Polymer Matrix	Mechanism of Action	Biological Activity	Ref.
Silver	Gelatin	Ion release, interaction with disulfide or sulfhydryl groups of enzymes that lead to the disruption of metabolic processes, DNA damage, disturbs the important cell functions.	*Salmonella typhimurium, Listeria monocytogenes, Escherichia coli, Staphylococcus aureus, and Bacillus cereus*	[126,156]
Silver	Chitosan/Starch		*Escherichia coli, Staphylococcus aureus*	[157]
Silver	Polyvinyl alcohol		*Salmonella typhimurium*	[158]
ZnO	Alginate	Ion release, generation of reactive oxygen species (ROS), membrane dysfunction.	*Salmonella typhimurium, Escherichia coli*	[126,159]
ZnO	Polyvinyl chloride		*Escherichia coli, Staphylococcus aureus*	[160]
TiO_2_	Chitosan		*Escherichia coli, Staphylococcus aureus*	[161]
TiO_2_	Ethylene vinyl alcohol	Oxidative stress through ROS, disrupts the cell integrity.	*Bacillus stearothermophilus, Staphylococcus aureus, Escherichia coli, Zygosaccharomices rouxii, Bacillus* sp., *Lactobacillus platarum, Erwinia caratovora, Pichia jadini, Pseudomonas fluorescens*	[126,162]
TiO_2_	Oriented-polypropylene		*Escherichia coli*	[163]
TiO_2_	Low-density polyethylene		*Rhodotorula mucilaginosa,Pseudomonas* spp.	[164]
CuO	Low-density polyethylene	Disturbs the vital enzymes of bacteria.	Coliforms	[165,166]
Eugenol encapsulated in mesoporous silica NPs	Poly(3-hydroxybutyrate co-3-hydroxyvalerate)	Essential oils (EO) and their active components disturb the structure and permeability of the cell membrane, where they interact with the proteins and enzymes and disturbs the important cell functions, for example, proton motive force, electron flow, energy regulation, or synthesis of structural components.	*Escherichia coli, Staphylococcus aureus*	[142,167,168,169]
Carvacrol-loaded HNTs	Polyethylene		*Aeromonas hydrophila*	[137]
EO components immobilized on silica particles			*Listeria innocua, Escherichia coli*	[141]
Carvacrol-loaded HNTs	Low-density polyethylene		*Listeria innocua, Escherichia coli, Alternaria alternata*	[135]
